# Omicron: A Blessing in Disguise?

**DOI:** 10.3389/fpubh.2022.875022

**Published:** 2022-05-02

**Authors:** Esayas Kebede Gudina, Solomon Ali, Guenter Froeschl

**Affiliations:** ^1^Department of Internal Medicine, Jimma University Institute of Health, Jimma, Ethiopia; ^2^Department of Microbiology, Immunology and Parasitology, St. Paul's Hospital Millennium Medical College, Addis Ababa, Ethiopia; ^3^Division of Infectious Diseases and Tropical Medicine, University Hospital, Ludwig-Maximilians-Universität, Munich, Germany; ^4^Center for International Health, Ludwig-Maximilians-Universität, Munich, Germany

**Keywords:** COVID-19, SARS-CoV-2, COVID vaccine, Omicron variant, vaccine inequalities, pandemic (COVID-19)

Two years after the first reported case and a year after the first shot of an effective vaccine, COVID-19 remains a major global threat and source of uncertainties. Although many thought a year ago that 2022 would be the year to return to normalcy, the world welcomed the New Year with record number of daily new cases in most countries. This is happening while we are having 10 vaccines approved for use by the World Health Organization (1) and some 64.5% of the global population has already taken at least a dose of one of the vaccines (2). The success against the pandemic was undermined by an inequitable distribution of the vaccines (3) and evolution of highly transmissible variants of the virus (4). Today while most of the wealthy countries have provided a booster dose vaccination for at least a third of their population (2), only 14% of the population in Africa has received the first shot (5). Paradoxically, there is a relevant proportion of the population, especially in high-income countries, that oppose getting immunized, as part of the no-vax movement (6).

The onset of November 2021 saw many countries relaxing their COVID-19 travel restrictions. However, the announcement of B.1.1.529 (Omicron), a highly mutated variant, by WHO on 26 November 2021 as variant of concern (7) led to an epidemiological situation that the world was not quite prepared for. This soon led to many countries reimposing their restrictions. Although mutations leading to new variants are evolutionary features of the virus (8), such occurrences remain a major setback even in an era where the world disposes of ever more tools to fight infectious diseases. The Delta variant (B.1.617.2) was first discovered in India in late 2020, spread to 179 countries and became the dominant variant globally in less than a year. It caused more infections, hospitalizations, and deaths globally specially among unvaccinated people than previous variants (9). This happened when there were effective vaccines already widely available.

Since its discovery in November 2021, the Omicron variant is spreading at an unprecedented rate, surpassing all previous variants (10). It is now the predominant variant circulating globally, due to its so far milder course of illness, and its potential to escape from vaccine-induced immune-responses (11). Omicron has several sub-lineages of which BA.1 and BA.2 are the most common ones (12). Although BA.1 has been the predominant Omicron sub-lineage until recently, the relative proportion of BA.2 sub-variant is increasing in several countries in the past few months (13). It is thought to be more transmissible and shorter doubling time than BA.1 (14). Existing evidence also shows that the BA.2 sub-variant has an even more pronounced immune escape capacity and higher resistance to existing treatments (15). Despite these facts, Omicron in general is associated with lower risk of severe disease, hospitalization (16), and death than the previous variants such as Delta (17).

Controlling the spread of Omicron has been found to be more challenging due to the diverse nature of the subvariants. Most infected people have milder symptoms and therefore may continue their social interactions, infecting many others in the process. The proportion of infected individuals ending up in hospitals and ICU as well as dying of COVID-19 may be lower than the previous circulating variants, but the absolute number may be much higher due to the sheer incidence of infections. Thus, the Omicron variant may ultimately result in a much higher pressure on public health systems than previous variants especially in resource limited settings. The surge of the new sub-lineages such as BA.2 may even prolong and aggravate the current Omicron wave (14).

On the other hand, the fact that this variant has milder symptoms may result in more people (vaccinated or unvaccinated) getting the infection with relatively lower health impact as per case. This may ultimately lead to widespread immunity in a faster way. Although the degree of protective immunity conveyed by natural infections from previous variants is not clear yet, recurrent infections and break-through infections in vaccinated people may lead to more robust immune responses (18).

The full picture of the upcoming months may reveal a high rate of transmission and at the same time a low proportion of severe disease and death. Hence, in a few months we may be able to approach some form of global herd immunity that would at least prevent severe diseases and death downstream, realizing the initial assumption that 2022 may become a year of return to normalcy, and SARS-CoV-2 becoming a member of the group of globally endemic flu-like infections (19).

## Global Surge of Ba. 2 Subvariant and the Looming Uncertainties

Although close to two third of the world population and over 80% of the population in the high-income countries have received at least one dose of COVID-19 vaccine (2), more daily new cases of COVID-19 are being reported globally than in the pre-vaccine era (20). The BA.2 subvariant, known to cause widespread infection even among vaccinated and previously infected individuals, is deriving the current wave (21). One of the features of this variant is the difficulty to track it with the current common tests and hence known as “Stealth Omicron”. Even though the standard real time PCR is able to detect BA.2, it may not be able distinguish it from the Delta variant (22). Thus, it may be underreported in settings where genomic sequencing is not performed routinely to track the variants.

While the full virological characteristics and epidemiology of the of BA.2 is still unfolding, it is spreading at an overwhelming rate than the previous variants (14). It is now the predominant variant globally and a cause of new peaks in countries with high vaccination coverage in Europe and Asia (20, 21). The lifting of COVID-19 restrictions in many countries has led to this recent surge due to BA.2.

Despite the unprecedented surge, this subvariant is not associated with more severe disease, hospitalization, and death than BA.1 and previous variants (16, 17). However, due to the waning immunity from vaccination and previous infections, and relaxation of most of the restrictions globally, it is possible to have another wave of the outbreak among unvaccinated population. Nevertheless, major health system crisis due to the outbreak is less likely to happen because of some form of immunity from vaccinations and previous infections (23).

Regardless of this optimism, the global action against the pandemic remains fragile as ever and mired with uncertainties. As we have seen in the past several months, new variants are evolving more frequently and BA.2 will not be the last one. As a result, SARS-CoV-2 remains a serious global public health issue and the world should remain vigilant to deal with the most likely new variants in the future. Boosting immunity against the virus through vaccination (24) and cutting its spread through non-pharmacological methods such as mask use (25) remain the most powerful and proven means to deal with the evolutionary adaptability of the virus.

The fight against the virus thus needs concerted global action through equitable distribution of vaccines, dealing with vaccine hesitancy, and optimizing non-pharmacological preventive interventions until the pandemic is under control at least to a degree that is not detrimental to health systems. The global community will benefit more from ensuring that as many people as possible are immunized globally, rather than from nation-states cocooning and stockpiling vaccines for their defined populations. This is true both from an ethical as well as from an epidemiological point of view. Countries should also put in place strategies to closely monitor and track SARS-CoV-2 emerging variants.

## Author Contributions

EG wrote the first draft. SA and GF reviewed the manuscript for intellectual contents. All authors have approved the manuscript in the current version.

## Conflict of Interest

The authors declare that the research was conducted in the absence of any commercial or financial relationships that could be construed as a potential conflict of interest.

## Publisher's Note

All claims expressed in this article are solely those of the authors and do not necessarily represent those of their affiliated organizations, or those of the publisher, the editors and the reviewers. Any product that may be evaluated in this article, or claim that may be made by its manufacturer, is not guaranteed or endorsed by the publisher.
